# Maternal Plasma 25-Hydroxyvitamin D Concentrations and the Risk for Gestational Diabetes Mellitus

**DOI:** 10.1371/journal.pone.0003753

**Published:** 2008-11-18

**Authors:** Cuilin Zhang, Chunfang Qiu, Frank B. Hu, Robert M. David, Rob M. van Dam, Alexander Bralley, Michelle A. Williams

**Affiliations:** 1 Division of Epidemiology, Statistics, and Prevention Research, Eunice Kennedy Shriver National Institute of Child Health and Human Development, National Institutes of Health, Bethesda, Maryland, United States of America; 2 Center for Perinatal Studies, Swedish Medical Center, Seattle, Washington, United States of America; 3 Department of Epidemiology, Harvard School of Public Health, Boston, Massachusetts, United States of America; 4 Department of Nutrition, Harvard School of Public Health, Boston, Massachusetts, United States of America; 5 Channing Laboratory, Department of Medicine, Brigham and Women's Hospital and Harvard Medical School, Boston, Massachusetts, United States of America; 6 Metametrix Clinical Laboratory, Norcross, Georgia, United States of America; 7 Department of Epidemiology, University of Washington School of Public Health and Community Medicine, Seattle, Washington, United States of America; Uppsala University, Sweden

## Abstract

**Background:**

Evidence is accumulating for a role of vitamin D in maintaining normal glucose homeostasis. However, studies that prospectively examined circulating concentrations of 25-hydroxyvitamin D (25-[OH] D) in relation to diabetes risk are limited. Our objective is to determine the association between maternal plasma 25-[OH] D concentrations in early pregnancy and the risk for gestational diabetes mellitus (GDM).

**Methods:**

A nested case-control study was conducted among a prospective cohort of 953 pregnant women. Among them, 57 incident GDM cases were ascertained and 114 women who were not diagnosed with GDM were selected as controls. Controls were frequency matched to cases for the estimated season of conception of the index pregnancy.

**Results:**

Among women who developed GDM, maternal plasma 25-[OH] D concentrations at an average of 16 weeks of gestation were significantly lower than controls (24.2 vs. 30.1 ng/ml, P<0.001). This difference remained significant (3.62 ng/ml lower on average in GDM cases than controls (P value = 0.018)) after the adjustment for maternal age, race, family history of diabetes, and pre-pregnancy BMI. Approximately 33% of GDM cases, compared with 14% of controls (*P*<0.001), had maternal plasma 25-[OH] D concentrations consistent with a pre-specified diagnosis of vitamin D deficiency (<20 ng/ml). After adjustment for the aforementioned covariates including BMI, vitamin D deficiency was associated with a 2.66-fold (OR (95% CI): 2.66 (1.01–7.02)) increased GDM risk. Moreover, each 5 ng/ml decrease in 25-[OH] D concentrations was related to a 1.29-fold increase in GDM risk (OR (95% CI): 1.29 (1.05–1.60)). Additional adjustment for season and physical activity did not change findings substantially.

**Conclusions:**

Findings from the present study suggest that maternal vitamin D deficiency in early pregnancy is significantly associated with an elevated risk for GDM.

## Introduction

Vitamin D, a secosteroid that is synthesized in skin and sequentially metabolized in liver and kidneys in humans, has been well-known for its function in maintaining calcium and phosphorus homeostasis and promoting bone mineralization. However, the ubiquitous distribution of intracellular vitamin D receptor across diverse tissues [1] and the emerging epidemiological evidence documenting increased risks of hypertension [2], [3], cardiovascular disease [4]–[7], and selected cancers [1], [8] associated with vitamin D deficiency underscore the pleiotropic actions of vitamin D. Evidence is also accumulating for a role of vitamin D in maintaining normal glucose homeostasis. For instance, in both animal and human studies, vitamin D depletion was significantly related to insulin resistance and impaired insulin secretion. Notably, this condition is reversible upon repletion of vitamin D [9]–[12]. Moreover, a significant and strong association between vitamin D deficiency and ß-cell dysfunction has been reported in healthy, non-diabetic, or diabetic populations [13]–[15]. Furthermore, circulating concentrations of 25-hydroxyvitamin D (25-[OH] D), the primary circulating form of vitamin D, were significantly and inversely related to the risk for type 2 diabetes[15] and related phenotypes in epidemiological studies [9], [16]–[19].

It has long been known that vitamin D deficiency is prevalent among pregnant women [20]–[22]. For instance, recent data among pregnant women in northern United States indicated that vitamin D deficiency occurred in 29.2% of black women and 5% of white women [23]. Data on the role of vitamin D in glucose homeostasis during pregnancy and the development of gestational diabetes mellitus (GDM) are scant and findings are inconsistent. In a cross-sectional study [24], serum concentrations of 25-[OH] D measured at the time of GDM screening test (24–28 weeks of gestation) were significantly lower in GDM women than in normal glucose tolerance pregnant women (16.49 vs. 22.97, *p* = 0.009). Similarly, in another study [25], maternal serum 25-[OH] D concentrations measured at the time of GDM screening test were significantly and inversely associated with fasting glucose. In an Indian population, no significant association between 25-[OH] D concentrations and GDM risk was observed [26]. Findings from these studies are interesting, inference is, however, hindered by ambiguous temporal relationship between circulating concentrations of 25-[OH] D and GDM risk due to their cross-sectional design. In the present study, we evaluated the association between maternal plasma vitamin D concentrations in early pregnancy and subsequent risk of GDM based on a prospective cohort study of pregnant women.

## Materials and Methods

### Study population

This nested case-control study was based on an ongoing prospective cohort study of pregnant women, the “Omega Study” [27]. In this cohort, participants were recruited from women attending prenatal care at clinics affiliated with Swedish Medical Center in Seattle and Tacoma General Hospital in Tacoma, Washington. Women were ineligible if they initiated prenatal care after 20 weeks gestation, were younger than 18 years of age, did not speak and read English, did not plan to carry the pregnancy to term, or did not plan to deliver at either of the two research hospitals. Participants completed a questionnaire administered in English by a trained interviewer at or near enrollment. These questionnaires were used to gather information on socio-demographic, anthropomorphic, and behavioral characteristics and reproductive and medical histories. After delivery, maternal and infant medical records were abstracted for information on the course and outcome of pregnancy. The procedures used in the Omega Study were in agreement with the protocols approved by the Institutional Review Boards of Swedish Medical Center and Tacoma General Hospital. All participants provided written informed consent.

The analytical population was selected from pregnant women who enrolled in the Omega Study between September 2002 and October 2004. During this period, a total of 953 pregnant women provided blood samples and completed interviews. Among them, we identified and sampled all 57 women who developed GDM and we randomly sampled 114 women who were not diagnosed with GDM as controls. Controls were frequency matched to cases for the estimated season of conception of the index pregnancy (i.e. spring, summer, autumn, winter).

### Data collection

From structured questionnaire and medical records, we obtained information of covariates including maternal age, educational attainment, height, pre-pregnancy weight, reproductive and medical histories, and medical histories of first-degree family members. We also collected information on annual household income and maternal smoking before and during pregnancy. Pre-pregnancy body mass index (BMI) was calculated as pre-pregnancy weight in kilograms divided by height in meters squared. Maternal medical records were reviewed to collect detailed medical and clinical information. In our study settings, according to the recommendations from the American Diabetes Association (ADA) [28], pregnant women were screened at 24–28 weeks gestation using a 50 gram 1-hour oral glucose challenge test. Those patients who failed this screening test (glucose≥7.8 mmol/L) were then followed-up within 1–2 weeks with a 100g, 3-h oral glucose tolerance test (OGTT). We also abstracted laboratory results from participants' 50 gram 1-hour glucose challenge test and from the diagnostic 100 gram 3-hour OGTT. Women were diagnosed with GDM if two or more of the 100 gram OGTT glucose levels exceeded the ADA criteria [28]: fasting >5.3 mmol/L; 1-hour >10.0 mmol/L; 2-hour >8.6 mmol/L; 3-hour >7.8 mmol/L.

Participants provided a 20 ml non-fasting blood sample around 16 weeks of gestation and reported the hours since last eating at the time of the blood draw. Immediately after blood samples were collected, they were fractionated by using standard procedures and stored at −80°C until analysis. Plasma 25-(OH) D concentrations were measured using DiaSorin enzyme immunoassay reagents and procedures (Metametrix, Norcross, GA). Intra-assay and inter-assay coefficients of variation for this method are both ≤12%.

### Statistical analysis

We estimated the correlation between plasma 25-[OH] D concentrations and maternal pre-pregnancy BMI using Pearson's correlation coefficients (r). Because the distribution of plasma 25-[OH] D concentrations was approximately normally distributed, we examined differences in mean concentrations between cases and controls using the Student's t test. We categorized plasma 25-[OH] D concentrations according to previously published criteria for vitamin D sufficiency (≥30 ng/ml), insufficiency (20–29 ng/ml) and deficiency (<20 ng/ml) [1]. We used logistic regression models to estimate odds ratios (OR) and 95% confidence interval (95% CI). We evaluated the covariates in Table 1 as potential confounders and included in the final model those that altered unadjusted ORs by 10% or more, including maternal age, race/ethnicity, family history of type 2 diabetes, and pre-pregnancy BMI. Inclusion of physical activity in the model only slightly changed the ORs (<3%), therefore, we didn't include it in the final model. All analyses were performed using Stata 9.0 (Stata, College Station, TX). All reported confidence intervals were calculated at the 95% level and all reported p-values are two-tailed.

**Table 1:** Characteristics of study participants according to gestational diabetes (GDM) case-control status.

Characteristics	GDM Cases	Controls	P-value
	(N = 57)	(N = 114)	
	n (%)	n (%)	
Maternal Age at interview (years)	34.3±4.8^1^	33.1±3.9	0.09
*<35*	29 (50.9)	72 (63.2)	0.12
*≥35*	28 (41.1)	42 (36.8)	
Maternal Race/Ethnicity			
*White/Non-Hispanic*	40 (70.2)	96 (84.2)	0.03
*African American*	2 (3.5)	6 (5.3)	
*Others*	15 (26.3)	12 (10.5)	
Nulliparous	27 (47.4)	62 (54.4)	0.39
Consumed Prenatal Vitamins	49 (86.0)	105 (92.1)	0.21
Consumed≥2 Servings of Fish Weekly	27 (47.4)	65 (57.0)	0.23
Consumed≥1 Glass of Milk Daily	22 (38.6)	42 (36.8)	0.82
Smoked During Pregnancy	3 (5.3)	7 (6.1)	0.82
Family History of Diabetes Mellitus	14 (24.6)	15 (13.2)	0.06
Physical Inactivity During Pregnancy	9 (15.8)	9 (7.9)	0.11
Pre-Pregnancy Body Mass Index (kg/m^2^)	26.7±7.2^1^	23.3±3.8	0.001
Pre-Pregnancy Body Mass Index (kg/m^2^)			
*<25*	29 (50.9)	84 (73.7)	0.003
*≥25*	28 (49.1)	30 (26.3)	
Gestational Age at Blood Collection (weeks)	16.3±2.1^1^	16.1±2.2	0.59
Gestational Age at Glucose Screening Test (weeks)	26.9±2.6^1^	26.4±2.5	0.25
Season of Estimated Time of Conception*			
*(March–May) Spring*	21 (36.8)	42 (36.8)	1.00
*(June–August) Summer*	17 (29.8)	34 (29.8)	
*(September–November) Autumn*	13 (22.8)	26 (22.8)	
*(December–February) Winter*	6 (10.5)	12 (10.5)	
Maternal Plasma 25-Hydroxyvitamin D Concentrations (ng/ml)			
*Mean±SD*	24.2±8.5^1^	30.1±9.7	<0.001

1 Mean±SD (standard deviation).

* Matched factor.

## Results

In general, women who developed gestational diabetes were older, heavier, more likely to have a positive family history of type 2 DM, and less likely to be Non-Hispanic White as compared with controls (Table 1). Moreover, the majority of study participants reported consuming prenatal multivitamins (86% in cases and 92% in controls, *P* for difference = 0.21). As expected, maternal plasma 25-[OH] D concentrations were inversely associated with maternal adiposity as estimated by pre-pregnancy BMI (ρ = −0.28, p = 0.04 in GDM cases; ρ = −0.25, *P* = 0.01 in controls).

**Figure 1:**
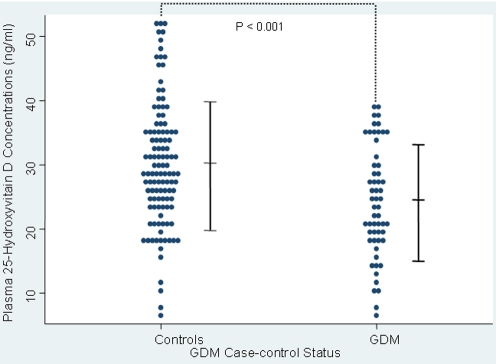
Maternal plasma 25-Hydroxyvitamin D concentrations in pregnancy among 57 GDM cases and 114 controls. Vertical bars indicate means±SD (standard deviation).

Maternal plasma 25-[OH] D concentrations were 20% lower, on average, among women who subsequently developed GDM, as compared with those who were not diagnosed with GDM (**Figure 1**). Notably, this difference in maternal plasma 25-[OH] D concentrations remained statistically significant (3.62 ng/ml lower on average in GDM cases than controls (*P* value = 0.018)) after the adjustment for maternal age, race/ethnicity, family history of diabetes, and pre-pregnancy BMI Similar findings were observed when we restricted our analyses to women without a history of GDM (mean±SD, GDM cases vs. controls: 24.9±8.2 *vs*. 29.7±9.4 ng/ml, *P* = 0.003).

Approximately 33% of GDM cases, compared with 14% of controls (*P*<0.001), had plasma 25-[OH] D concentrations consistent with a diagnosis of vitamin D deficiency (<20 ng/ml)) [1] **(** **Table 2** **)**. Women classified as being deficient for vitamin D had a 3.7-fold increased subsequent risk of GDM, as compared with vitamin D sufficient women (≥30 ng/ml) after adjustment for maternal age, race/ethnicity, first-degree family history of type 2 diabetes (adjusted OR = 3.74, 95% CI, 1.47–9.50). In addition, we analyzed plasma 25-[OH] D concentrations as continuous variables. From the multivariate analysis, each 5 ng/ml decrease in plasma 25-[OH] D concentrations was associated with a 1.36-fold (95% CI, (1.11–1.69)) reduction in GDM risk. Further adjustment for maternal pre-pregnancy BMI attenuated the association, though, it remained statistically significant. When we restricted this analysis to Non-Hispanic Whites, the majority of our study participants (40 GDM cases and 96 controls), 25-[OH] D deficiency was associated with an increased risk after the adjustment for BMI and other covariates (OR = 3.77, 95% CI 1.19–11.9). Among Non-Hispanic Whites, each 5 ng/ml decrease in plasma 25-[OH] D concentration was associated with a 1.29-fold increase in GDM risk (adjusted OR (95% CI), 1.29 (1.05–1.69)).

**Table 2:** Odds ratios (OR) and 95% confidence intervals (CI) for gestational diabetes (GDM) according to maternal plasma 25-hydroxyvitamin D (25(OH)D) concentrations in pregnancy.

25(OH)D (ng/ml)	GDM Cases	Controls	Unadjusted	Adjusted*	Adjusted**
	(N = 57)	(N = 114)	OR (95% CI)	OR (95% CI)	OR (95% CI)
**All Subjects**					
*25(OH)D (categorical variable)*					
Sufficient (≥30)	14	51	1.00 (referent)	1.00 (referent)	1.00 (referent)
Insufficient (20–29)	24	47	1.86 (0.86–4.01)	1.86 (0.84–4.09)	1.56 (0.69–3.52)
Deficient (<20)	19	16	4.33 (1.78–10.5)	3.74 (1.47–9.50)	2.66 (1.01–7.02)
P for trend			0.001	0.006	0.05
*25(OH)D(continuous variable)*					
Per 5 ng/ml reduction			1.44 (1.16–1.69)	1.36 (1.11–1.69)	1.29 (1.05–1.60)
**Non-Hispanic Whites**					
*25(OH)D (categorical variable)*					
Sufficient (≥30)	11	45	1.00 (referent)	1.00 (referent)	1.00 (referent)
Insufficient (20–29)	16	42	1.56 (0.65–3.74)	1.58 (0.65–3.87)	1.21 (0.47–3.09)
Deficient (<20)	13	9	5.91 (2.02–17.3)	5.40 (1.78–16.4)	3.77 (1.19–11.9)
P for trend			0.002	0.005	0.04
*25(OH)D (continuous variable)*					
Per 5 ng/ml reduction			1.44 (1.16–1.79)	1.44 (1.11–1.79)	1.29 (1.05–1.69)

Vitamin D deficiency was defined using cut-points given by Holick, MF ([reference 1]).

* Adjusted for maternal age, race/ethnicity and family history of diabetes.

** Adjusted for maternal age, race/ethnicity and family history of diabetes as well as pre-pregnancy body mass index.

We further examined the independent and joint effect of maternal plasma 25-[OH] D concentrations and overweight status on the risk of GDM. The association of GDM with 25-[OH] D concentrations was similar for overweight (BMI≥25 kg/m^2^) and lean women (BMI <25 kg/m^2^) (P for interaction 0.93). The risk for GDM was highest for overweight women who were classified as being deficient for vitamin D (25-[OH] D<20 ng/ml in pregnancy); they experienced an approximately 5-fold increased risk as compared with lean women of higher plasma 25-[OH] D levels (adjusted OR = 4.93, 95% CI 1.63–14.9) (**Table 3**).

**Table 3:** Odds ratios (OR) and 95% confidence intervals (CI) for gestational diabetes (GDM) according to both maternal plasma 25-Hydroxyvitamin D deficient status and pre-pregnancy overweight status.

Vitamin D deficiency*	Overweight**	GDM Cases	Controls	Unadjusted OR (95% CI)	Adjusted^1^ OR (95% CI)
		(N = 57)	(N = 114)		
No	No	22	75	1.00 (referent)	1.00 (referent)
Yes	No	7	9	2.65 (0.89–7.93)	2.36 (0.75–7.43)
No	Yes	16	23	2.37 (1.07–5.25)	2.24 (0.99–5.08)
Yes	Yes	12	7	5.84 (2.05–16.6)	4.93 (1.63–14.9)
*P value for interaction term*				*0.93*	*0.93*

* Vitamin D deficiency is defined as maternal plasma 25-Hydroxyvitamin D concentrations <20 ng/ml (Holick MF, [reference 1]).

** Overweight is defined as pre-pregnancy body mass index (BMI) ≥25 kg/m^2^.

1 Adjusted for maternal age, race/ethnicity, and family history of diabetes.

## Discussion

In the present study, maternal plasma 25[OH] D concentrations in early pregnancy were significantly and inversely associated with GDM risk. This association remained statistically significant even after controlling for established risk factors of GDM including maternal age, family history of type 2 diabetes, race/ethnicity, and pre-pregnancy BMI.

The major sources of vitamin D in the body are dietary vitamin D intake and supplementation as well as endogenous production of vitamin D in the skin exposed to sunlight. The biologically active form of vitamin D is 1,25-[OH] D. However, 25-[OH] D is regarded as the best indicator of vitamin D status in the body because it is the substrate for the renal and nonrenal production of 1,25-[OH] D and has a longer biological half-life and higher concentrations in circulation than 1,25-[OH] D. Plasma 25-[OH] D reflects vitamin D from both endogenous and exogenous sources.

It has become increasingly clear that vitamin D has physiological functions beyond bone health [6]; vitamin D receptors were expressed in a large number of other tissues including those involved in the regulation of glucose metabolism, such as muscle and pancreatic β cells [1]. GDM was hypothesized to result from pregnancy induced insulin resistance and impaired insulin secretion to compensate for it [28], [29]. Several mechanisms may explain the observed association between vitamin D deficiency and GDM risk. First, vitamin D may directly or indirectly modulate pancreatic β-cell function and secretion by binding its circulating active form, 1,25-[OH]D, to β-cell vitamin D receptor and regulating the balance between the extracellular and intracellular β-cell calcium pools [12], [30]. Second, vitamin D can promote insulin sensitivity by stimulating the expression of insulin receptors and enhancing insulin responsiveness for glucose transport. It also regulates extracellular calcium and thus ensures normal calcium influx through cell membranes and **an** adequate intracellular cytosolic calcium pool, which is essential for insulin-mediated intracellular processes in insulin-responsive tissues [31]. Lastly, it is possible that the observed inverse association of plasma 25-[OH] D with GDM risk reflects the impact of other components of major endogenous and exogenous sources of vitamin D on glucose homeostasis through other pathways. For instance, endogenous production of vitamin D in the skin due to sun exposure is a major source of plasma 25-[OH] D. Sun exposure could be positively correlated with outdoor physical activity, a protective factor for insulin resistance and GDM [32]–[34]. In the present study, the inverse association between plasma 25-[OH] D concentrations and GDM risk persisted after controlling for physical activity.

Data relating vitamin D to the risk for GDM are sparse. Our findings are in line with those from one cross-sectional study, where serum concentrations of 25[OH] D levels during 24–28 weeks of gestation were significantly lower in GDM women than in normal groups. [24] In another study [35], maternal serum 25-[OH] D concentrations measured at the time of GDM screening test were significantly and inversely associated with fasting glucose, although the association with GDM risk was not statistically significant. In a study among an Indian population, no significant association between 25-[OH] D concentrations (30 weeks of gestation) and GDM risk was observed [26]. Findings from these studies are interesting, inference is, however, hindered by ambiguous temporal relationship between circulating concentrations of 25-[OH] D and GDM because 25-[OH] D concentrations were measured in late pregnancy when GDM likely developed.

Our findings are also generally consistent with a relatively large body of literature documenting a significant and inverse association of circulating vitamin D concentrations with measurements of glycemia or presence of type 2 DM among non-pregnant women, although the majority of these findings are based on cross-sectional/retrospective data. For instance, serum 25-[OH]D concentrations (after multivariate adjustment) were inversely associated with the prevalence of diabetes in a dose-dependent pattern in both non-Hispanic Whites and Mexican-Americans in a large cross-sectional study [18]. Moreover, in a meta-analysis of cross-sectional studies [36], the summary OR for type 2 DM was 0.54 (95% CI, 0.23–1.27) for the highest vs. the lowest 25-[OH]D concentrations (25–38 *vs*. 10–23 ng/ml, respectively). When the data on Non-Hispanic Blacks were excluded, 25-[OH]D concentrations were significantly and inversely associated with prevalent type 2 DM [OR 0.36 (95% CI, 0.16–0.80)]. In the present study, when we restricted this analysis to Non-Hispanic Whites, the majority of our study participants, 25-[OH] D deficiency was associated with 3.77-fold increased risk after the adjustment for covariates. We do not have sufficient statistical power to examine the association in other race/ethnicity groups. We are aware of only one prospective study for type 2 DM thus far; an inverse association of serum 25-[OH]D with the incidence of type 2 DM was observed in a Finnish study [37]. Similarly, an inverse association of baseline serum 25-[OH]D concentration with future glucose levels and insulin resistance was observed in a recent population-based study of non-diabetic men and women aged 40–69 years [38].

Our study has several strengths. First, determination of 25-[OH] D concentrations using plasma collected in early pregnancy served to define the temporal relationship between maternal vitamin D deficiency and subsequent risk of GDM because, in general, pregnant women develop gestational diabetes during the late 2^nd^ or 3^rd^ trimester. We, however, cannot completely exclude the plausibility that a limited number of women may have had undiagnosed pre-pregnancy glucose intolerance when blood specimens were collected. Second, the high follow-up rate (>95%) of women enrolled in our study minimized possible selection bias. However, several limitations also merit discussion and consideration. First, a single measurement of plasma 25-[OH] D concentrations is not likely to provide a time-integrated measure of maternal vitamin D status during the entire study pregnancy. Longitudinal studies with serial measurements of maternal plasma 25-[OH] D concentrations, indices of insulin sensitivity and secretion are needed to elucidate the mechanisms and patho-physiological consequences of maternal vitamin D deficiency during pregnancy. Second, our study is relatively small and thus, large prospective studies are needed to confirm our results. A further limitation is the under representation of minority women in our study populations. However, due to the predisposition of Asian, Black, and Hispanic women for insulin resistance and vitamin D deficiency, it is possible that adequate vitamin D status may be even more important in these populations.

In conclusion, our study provides data indicating that maternal vitamin D deficiency in early pregnancy (i.e. plasma 25-[OH] D <20 ng/ml) is significantly associated with elevated risk for GDM even after adjustment for conventional risk factors for diabetes. Such evidence is valuable in view of the limited prospective data on the relation of circulating vitamin D status to impaired glucose tolerance in either pregnant or non-pregnant individuals. Vitamin D deficiency is common among pregnant women and has been associated with elevated risk for other pregnancy complications such as preeclampsia and a number of serious short- and long-term health problems in offspring [20], [39]. Circulating vitamin D can be modified by food consumption (e.g. fatty fish), supplement use, and outdoor sun exposure. Intervention studies [14], [40]–[44] assessing the effect of vitamin D and calcium supplements on glucose metabolism among non-pregnant individuals have yielded mixed results, which is at least partly due to variations in the dose of supplementation and the duration of follow up. Optimizing the effective dose of vitamin D supplement continues to be a challenge. If confirmed, our data raise the possibility that treating vitamin D deficiency in pregnancy, via supplementation or lifestyle measures, can contribute to the prevention of GDM.
